# microRNA expression profiling identifies a four microRNA signature as a novel diagnostic and prognostic biomarker in triple negative breast cancers

**DOI:** 10.18632/oncotarget.1682

**Published:** 2014-01-21

**Authors:** Pierluigi Gasparini, Luciano Cascione, Matteo Fassan, Francesca Lovat, Gulnur Guler, Serdar Balci, Cigdem Irkkan, Carl Morrison, Carlo M. Croce, Charles L. Shapiro, Kay Huebner

**Affiliations:** ^1^ Department of Molecular Virology, Immunology and Medical Genetics, Ohio State University Wexner Medical Center and Comprehensive Cancer Center, Columbus, Ohio, USA; ^2^ ARC-NET Research Centre, University and Hospital Trust of Verona, Verona, Italy; ^3^ Department of Pathology, Hacettepe University, Ankara,Turkey; ^4^ Department of Pathology, Yildirim Beyazit University, Ankara Ataturk Research and Training Hospital, Ankara, Turkey; ^5^ Dr. Abdurrahman Yurtaslan Ankara Oncology Training and Research Hospital of the Ministry of Health; ^6^ Department of Pathology, Roswell Park Cancer Institute, S606 Basic Science Building, Elm and Carlton Streets Buffalo, NY; ^7^ Division of Medical Oncology and the Breast Program James Cancer Hospital and Ohio State University Comprehensive Cancer Center, Columbus, Ohio, USA; ^8^ IOR, Institute of Oncology Research, Bellinzona, Switzerland

**Keywords:** Triple Negative breast cancer, microRNA, five markers, prognosis, treatments, outcome

## Abstract

Triple Negative Breast Cancers (TNBC) is a heterogeneous disease at the molecular and clinical level with poor outcome. Molecular subclassification of TNBCs is essential for optimal use of current therapies and for development of new drugs. microRNAs (miRNA) are widely recognized as key players in cancer progression and drug resistance; investigation of their involvement in a TNBC cohort may reveal biomarkers for diagnosis and prognosis of TNBC. Here we stratified a large TNBC cohort into Core Basal (CB, EGFR and/or CK5, 6 positive) and five negative (5NP) if all markers are negative. We determined the complete miRNA expression profile and found a subset of miRNAs specifically deregulated in the two subclasses.

We identified a 4-miRNA signature given by miR-155, miR-493, miR-30e and miR-27a expression levels, that allowed subdivision of TNBCs not only into CB and 5NP subgroups (sensitivity 0.75 and specificity 0.56; AUC=0.74) but also into high risk and low risk groups. We tested the diagnostic and prognostic performances of both the 5 IHC marker panel and the 4-miRNA expression signatures, which clearly identify worse outcome patients in the treated and untreated subcohorts. Both signatures have diagnostic and prognostic value, predicting outcomes of patient treatment with the two most commonly used chemotherapy regimens in TNBC: anthracycline or anthracycline plus taxanes. Further investigation of the patients' overall survival treated with these regimens show that regardless of IHC group subdivision, taxanes addition did not benefit patients, possibly due to miRNA driven taxanes resistance. TNBC subclassification based on the 5 IHC markers and on the miR-155, miR-493, miR-30e, miR-27a expression levels are powerful diagnostic tools. Treatment choice and new drug development should consider this new subtyping and miRNA expression signature in planning low toxicity, maximum efficacy therapies.

## INTRODUCTION

Triple-negative breast cancers (TNBC), defined by the absence of estrogen receptor, progesterone receptor, and HER-2 expression, account for 12% to 24% of all breast cancers. TNBCs are associated with early recurrence of disease and poor outcome.

Through gene expression profiling, six different TNBC subtypes, defined by abrogation of signaling pathways, have been identified: basal-like 1 and 2 (BL1 and BL2), immunomodulatory, mesenchymal, mesenchymal stem-like, and luminal androgen receptor-expressing [1]. These molecular entities have shown significant differences in terms of incidence, risk factors, prognosis and response to treatment [1-3].

Approximately 15% of breast cancers are basal-like and are associated with poor relapse-free and overall survival [4-6]. The basal-like subtype is of particular clinical interest due to its high frequency, lack of effective targeted therapies, poor baseline prognosis, and tendency to affect younger women. Over the years, basal-like breast cancer has become commonly known as triple-negative breast cancer (TNBC), lacking estrogen receptor (ER) and progesterone receptor (PR) expression as well as human epidermal growth factor receptor 2 (HER2) amplification; however, not all TNBCs are identified as basal-like by gene expression, and not all basal-like tumors are Triple Negative (TN) [7]. Subclassification is necessary to better identify molecular-based therapeutic targets, select biomarkers, discover new drugs, and design clinical trials that will enable alignment of TNBC patients to appropriate targeted therapies. Cost and complexity issues can render gene expression profiling impractical as a routine hospital diagnostic tool, while immunohistochemical (IHC) marker detection is feasible for the majority of institutions.

Nielsen et al (2004) [8] and Carey et al (2006) [9] showed that detection of epidermal growth factor receptor (EGFR) and/or cytokeratin 5/6 (CK5/6) expression by IHC staining can accurately identify the basal-like tumors among cohorts profiled by expression microarray with 100% specificity and 76% sensitivity [6]. This ‘five-marker panel’ ER-PR-HER2–EGFR-CK5/6 allows subclassification of TNBCs as basal-like (or Core Basal, CB) when EGFR and/or CK5/6 are positive or five negative (5NP) if all markers are negative. In our current study, we have correlated the 5 marker (ER-PR-HER2-EGFR-CK5/6) IHC expression profiles with microRNA (miRNA) expression and generated a four-miRNA prognostic signature that stratifies with high specificity CB and 5NP by overall survival. We also have correlated the miRNA signature with prognosis and survival following treatment with different chemotherapy regimens.

MiRNAs are 19–25 nucleotide, non-coding RNAs that reduce the abundance and translational efficiency of mRNAs and play a major role in regulatory networks, influencing diverse biological processes [10, 11] through effects of individual miRNAs on translation of multiple mRNAs. We previously determined miRNA expression profiles and expression profiles of a cancer-focused mRNA panel, in breast cancers, adjacent non-tumor (normal) and lymph node metastatic lesion (mets) tissues, from 173 women with TNBCs; we then linked specific miRNA signatures to patient survival and used miRNA/mRNA anti-correlations to identify clinically and genetically different TNBC subclasses.

For the current study we stratified the TNBC cohort based on EGFR and CK5/6 scoring, into CB and 5NP subgroups and determined if there is a subset of miRNAs specifically deregulated in one or the other of the two subclasses. We indeed found a four miRNA signature which allowed subdivision of TNBCs into two subgroups (high risk and low risk) for which associations among specific clinical features and the four miRNAs were then sought, including outcomes based on specific chemotherapy regimens.

The role of specific chemotherapy agents in the treatment of TNBC remains incompletely defined. Taxanes and anthracyclines are active in TNBC and remain important agents, but have not shown specific benefit for TNBC patients *vs* non-TNBC [12, 13]. Although TNBC is associated with a poor prognosis, some patients respond well to anthracycline-based chemotherapy, reflecting a significant degree of molecular heterogeneity within this subgroup [14-16].

Thus, we have examined and compared the prognostic value of the IHC based subclassification in CB and 5NP and of a four miRNA signature efficacy relative to specific chemotherapy regimens.

## RESULTS

### Definition of triple negative breast cancer biological subtypes by immunohistochemistry

Two TMAs comprehensive of the TNBC cohort profiled for miRNA expression [17] were evaluated by immunohistochemical analysis to assess the expression of ER, PR, HER2, EGFR, and CK5/6. FISH analysis for the *HER2* gene was also performed, with no gene amplification observed in all the tested cases.Cases were categorized based on their IHC profiles into two subclasses: (I) triple-negative cancers (i.e. ER-PR-HER2 negative) expressing EGFR and/or CK5/6, here referred to as CB, the so called “basal-like” as defined by mRNA expression analysis; and (II) cancers negative for the five markers, referred to as the 5NP subclass, triple negative cancers that express neither EGFR nor CK5/6, or “non basal” if considering the definition by mRNA expression.Of the 160 TNBCs considered, 82 were negative for EGFR (51%), and 131 for CK5/6 (82%). A total of 92 cases (57.5%) were classified as CB, and 68 (42.5%) as 5NP. The clinico-pathological characteristics of the entire TNBC cohort, as well as of the two subclasses, are summarized in Table 1.

**Table 1:** Clinical and demographic characteristics of the TNBC cohort.

Characteristics	Entire cohort (n=173)	IHC (n=160)		miRNA signature (n=160)	
		CB	5NP	High Risk	Low Risk
Number of cases		92 (57.5%)	68 (42.5%)	80 (50%)	80 (50%)
Race					
Caucasian	153	82 (89.1%)	61 (89.7%)	71 (88.6%)	72 (90%)
African American	16	7 (7.6%)	7 (10.3%)	7 (8.8%)	7 (8.8%)
Other	4	3 (3.3%)	0 (0%)	2 (2.6%)	1 (1.2%)
Menopause status					
Pre-menopausal	64	35 (38%)	21 (31%)	31 (38.8%)	25 (31.2%)
Post-menopausal	103	54 (58.7%)	46 (67.6%)	47 (58.6%)	53 (66.2%)
Unknown	6	3 (3.3%)	1 (1.4%)	2 (2.6%)	2 (2.6%)
Grade					
I	2	1 (1.2%)	0 (0%)	1 (1.2%)	0 (0%)
II	15	6 (6.5%)	8 (11.8%)	9 (11.2%)	5 (6.2%)
III	150	83 (89.1%)	59 (88.2%)	67 (83.8%)	75 (93.8%)
Unknown	6	2 (2.2%)	1 (1.4%)	3 (3.8%)	0 (0%)
LN metastases					
Positive	62	36 (39.1%)	22 (32.4%)	28 (35%)	30 (37.4%)
Negative	102	50 (54.4%)	43 (63.2%)	45 (56.2%)	48 (60%)
Unknown	9	6 (6.5%)	3 (4.4%)	7 (8.8%)	2 (2.6%)
Age at diagnosis					
<=40	34	17 (18.5%)	14 (20.6%)	16 (20%)	15 (18.7%)
41-50	52	30 (32.6%)	13 (19.2%)	27 (33.8%)	16 (20%)
>=51	87	45 (48.9%)	41 (60.2%)	37 (46.2%)	49 (61.3%)
Death					
No	114	57 (62%)	48 (70.6%)	43 (53.7%)	62 (78.7%)
Yes	59	35 (38%)	20 (29.4%)	37 (46.3%)	18 (21.3%)
*Recurrence					
No	126	63 (68.5%)	53 (77.9%)	53 (66.2%)	63 (78.7%)
Yes	47	29 (31.5%)	15 (22.1%)	27 (33.8%)	17 (21.3%)
*Type of 1st recurrence					
In situ	1	1 (1.2%)	0 (0%)	1 (1.2%)	0 (0%)
Local Regional	3	2 (2.2%)	1 (1.4%)	2 (2.5%)	1 (1.2%)
Distant	35	23 (25%)	12 (17.8%)	21 (26.3%)	14 (17.5%)
Type unknown	8	3 (3.3%)	2 (2.9%)	3 (3.8%)	2 (2.6%)
Chemotherapy					
None	25	11 (12%)	11 (16.2%)	10 (12.5%)	12 (15%)
Anthracycline. (primarily AC. FEC)	32	13(14.1%)	16 (23.5%)	12 (15%)	17 (21.3%)
Anthracycline + taxanes (AC + taxol + taxotere)	36	25 (27.1%)	9 (13.3%)	20 (25%)	14 (15.5%)
Non anthracycline. no taxanes (CMF)	9	6 (6.5%)	2 (2.9%)	4 (5%)	4 (5%)
Taxane(s) + non anthracycline drugs	5	3 (3.3%)	2 (2.9%)	4 (5%)	1 (1.2%)
Not Available info (NA)	66	34 (37%)	28 (41.2%)	30 (37.5%)	32 (40%)

**Tab. Abbreviations:** CB (Core Basal); 5NP (five negative phenotype); AC, doxorubicin and cyclophosphamide; CMF, cyclophosphamide, methotrezate, and fluorouracil; FEC fluorouracil, epirubicin, cyclophosphamide; FAC, fluorouracil, doxorubicin, and cyclophosphamide. Percentages are referred within the subtype. 1= anthracycline containing regimen

### miRNA expression profiles of the CB and 5NP subclasses identifies a diagnostic four miRNA signature

**FIGURE 1 (A, B, C):**
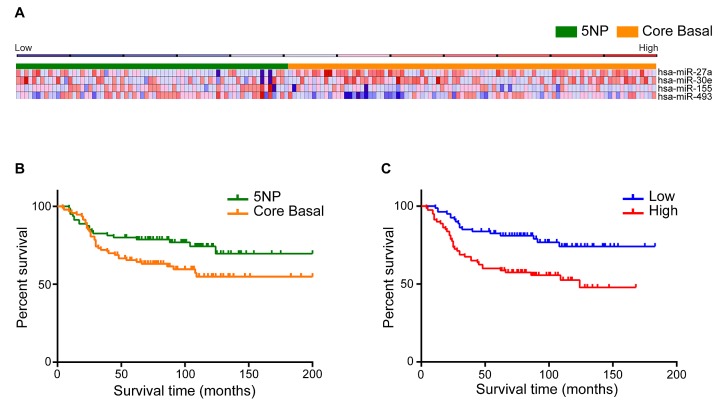
Identification of miRNAs that are differentially expressed in CB and 5NP breast cancers A) Heat map representing miRNA profiles of 160 tumor samples using complete linkage and Pearson correlation method as distance metrics. Orange identifies CB and Green 5NP tumors. Columns represent individual cancers; rows represent expression of miRNAs. Heat map colors represent relative miRNA expression as indicated in the blue to red key bar at the top. B) Overall survival of 160 TNBC tumors based on the status of the five IHC markers. C) COX proportional hazard model shows the overall survival based on the four miRNA high / low risk signature.

To identify diagnostic miRNA signatures in TNBCs, miRNA expression profiles already analyzed for TNBC expression pattern (Supplementary Figure 1), were examined to find differently deregulated miRNAs among CB and 5NP tumors.Supervised clustering of the cohort based on the IHC results (Figure 1A) shows a signature of four miRNAs that performed best in differentiating between CB and 5NP cancers (Figure 1A and Supplementary Table 1): miR-155 (logFC 0.76; p=0.04), miR-493 (logFC 0.54; p=0.01), miR-30e (logFC -0.61; p=0.04), and miR-27a (logFC -0.80; p=0.01). This four miRNA signature displayed sensitivity 0.75 and specificity 0.56 (AUC=0.74) in subclassifying CB or 5NP.

### miRNA signature impacts survival of TNBCs

Based on IHC subtyping, CB cancers exhibited a significantly worse outcome than 5NP (HR=1.76; 95% CI=1.04-2.92; p=0.03) (Figure 1B). Upregulation of miR-155 (HR=0.73; 95% CI=0.57–0.92) and of miR-493 (HR=0.88; 95% CI=0.72–0.99) correlated with better patient outcome so were defined as “protective”; down-regulation of miR-30e (HR=1.08; 95% CI=1.03–1.79) and of miR-27a (HR=1.09; 95% CI=1.03–1.79, correlated with a worse outcome so were defined as “risk”-associated (Supplementary Table 2).Cox proportional hazards models were applied to find, significant associations of the four deregulated miRNAs with CB and 5NP patient outcomes. All tumors were classified into high- or low-risk groups according to their risk-score (see Materials and Methods). The Kaplan-Meier overall survival (OS) graph, according to the combined four deregulated miRNAs is shown in Figure 1C. The median OS for the high *vs* low risk miRNA signature were 75.5 *vs* 82 months (HR=2.46; 95% CI=1.43-4.12; p=0.001), indicating a significant association between expression of the miRNAs and OS.

### Prognostic impact of TNBC subtype classification dependent on specific therapy regimens

**FIGURE 2 (A, B, C, D):**
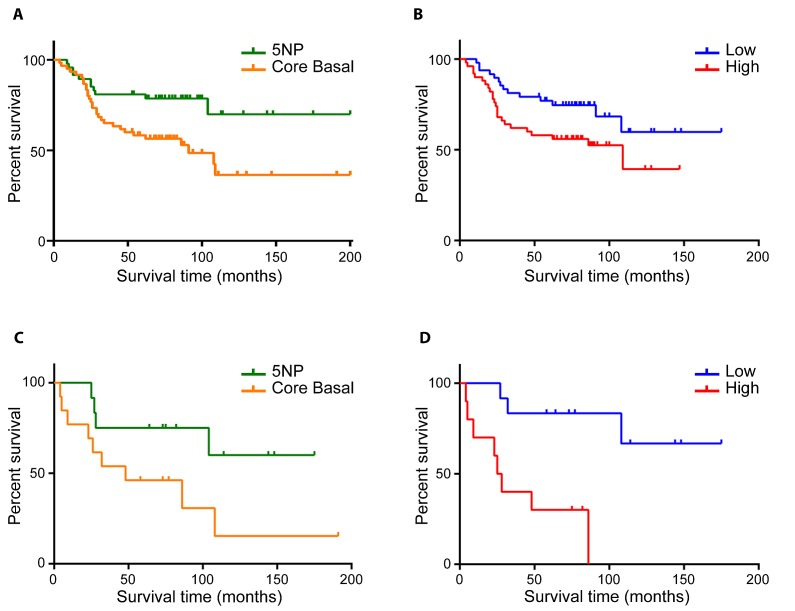
Overall survival of chemotherapy-treated and untreated TNBC patients A) IHC based overall survival of CB *vs* 5NP patients receiving chemotherapy; B) COX proportional hazard survival model of chemotherapy-treated patients stratified by high/low risk 4 miRNA signature C) Overall survival of IHC-stratified CB and 5NP untreated patients D) COX proportional hazard survival model of untreated patients based on 4 miRNA signature predictor

Since CB and 5NP subclasses have distinct OS as well high/low risk miRNA signature-based, we next analyzed the correlations among: subclasses, chemotherapy regimens and outcome.In the 107 patients that received chemotherapy, the CB group had significantly worse OS compared to the 5NP group (HR=2.46; 95% CI = 1.25-4.25; p=0.008) (Figure 2A). A similar trend was observed when stratifying the TNBCs for high and low risk by the miRNA signature (Figure 2B), the high-risk subgroup having a lower OS compared to the low-risk group (HR=1.98; 95% CI = 1.04-3.74; p=0.04).

In patients not undergoing chemotherapy, both IHC and four miRNA signature subtyping significantly stratified patients in prognostic classes (Figures 2C and 2D). 5NP untreated patients showed a better outcome compared to the untreated CB group (HR=2.97; 95% CI=1-8.87; p=0.05). The four miRNA signature subclassification performed better in discriminating high risk patients in comparison to the IHC subtyping (HR=6.19; 95% CI = 2.45-32.16; p=0.001).If the group of patients for whom chemotherapy treatment information was not available (NA) are stratified according to IHC status there is a clear trend toward worse survival of the CB subtype (Supplementary Figure 2A); also the four miRNA predictor can even more clearly separate this cohort into high/low risk cancers (HR=4.07; 95% CI = 1.44-9.75; p=0.007) (Supplementary Figure 2B).

TNBC patients are usually treated with a regimen of anthracycline or anthracycline plus taxanes, regardless of their proven intrinsic heterogeneity or IHC status. As shown in Table 1, within the entire TNBC cohort, four main chemotherapeutic regimens were administered: 1- anthracycline containing regimen (primarily doxorubicin and cyclophosphamide [AC], fluorouracil, epirubicin, cyclophosphamide [FEC]); 2- anthracycline + taxanes (AC+ taxol or taxotere); 3- non-anthracycline, no taxane containing regimen (cyclophosphamide, methotrexate, fluorouracil [CMF]); 4- taxane alone or in combination with non-anthracyline drugs (primarly taxol alone).

Among the 92 CB TNBC patients, 11 received no chemotherapy, 38 were treated with anthracycline-based chemotherapy (13 primarily with doxorubicin/cyclophosphamide and 25 with doxorubicin, taxol, taxotere) and 9 were treated with non-anthracycline-based chemotherapy (6 with cyclophosphamide, methotrexate, fluorouracil and 3 taxane or taxanes). Among the 68 5NP TNBC patients, 11 received no adjuvant systemic therapy, 15 received anthracycline-based chemotherapy (6 primarily doxorubicin/cyclophosphamide and 9 doxorubicin, taxol, taxotere), and 2 received non-anthracycline-based chemotherapy (Table 1).

**FIGURE 3 (A, B, C):**
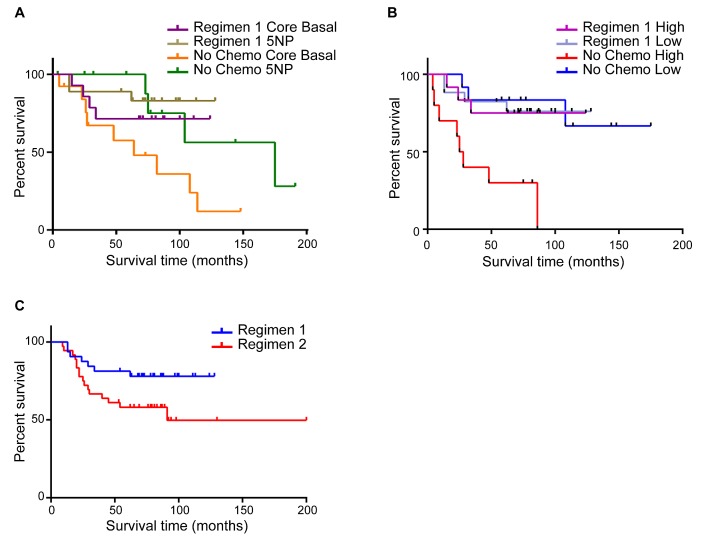
Overall survival of TNBC patients according to different chemotherapy regimens A) Overall Survival of CB and 5NP IHC-defined patients treated with regimen 1 chemotherapy or untreated; B) COX proportional hazard model of overall survival of patients treated with regimen 1 chemotherapy or untreated, stratified by high/low risk 4 miRNA signature; C) Overall survival of all patients treated with chemotherapy regimen 1 or regimen 2.

We stratified the cohort into the CB and 5NP subclasses and then considered anthracycline containing regimen *vs* untreated patients (Figure 3A). Besides the not unexpected positive effect of the chemotherapy in both subclasses, it is observed that the untreated 5NP group showed longer life expectancy than the chemotherapy treated CB group (p=0.027). This finding underlines the much better prognosis of the 5NP *vs* the CB cancers regardless of chemotherapy, as well as the lower efficacy of this therapy for the 5NP group. The high/low risk miRNA signature overall survival shown in Figure 3B is comparable to the IHC-based (Figure 3A), where the anthracycline-containing regimen significantly influenced the prognosis of high-risk tumors (p<0.001). On the other hand, there is little if any effect of the anthracycline-containing regimen in the low risk patient group.

Considering that anthracycline-containing regimen results in the best outcome regardless of stratification by IHC or high/low miRNA signature status (data not shown) we proceeded with the investigation of anthracycline plus taxanes Regimen 2. Conflicting data have been reported on the specific benefit of taxanes for adjuvant therapy in TNBC. The analysis of our cohort gave interesting results for consideration. Regardless of the IHC status, the addition of taxanes apparently does not improve OS, and may worsen the prognosis (Figure 3C). Because the number of patients treated within the Regimen 2 was too small (34 CB vs 9 5NP) to have a solid statistics about the IHC and miRNA signature performances, we considered different possible scenarios about this observation. The intriguing finding concerning the efficacy of taxanes can be explained by investigating the expression of Vimentin, a protein that has been linked to taxanes resistance in breast cancer and considered a marker for adverse prognosis [18-21]. We evaluated Vimentin expression by IHC in the whole cohort and we found that was up-regulated in CB tumors (CB, 43.5% positive; 5NP, 27.9% positive; p=0.044); this is in accordance with several studies that correlate Vimentin upregulation with taxanes resistance and worst patient prognosis.

Another possible scenario is that Regimen 2 treated patients might present a higher population of breast cancer stem cells (BCSCs) identified by the cell-surface markers CD44^+^/CD24^−/low^ which demonstrate chemo- and radiotherapy resistance [22-24]. From a first analysis of CD44^+^/CD24^−/low^ protein stain on the associated TMA of the cohort, we couldn't find any correlation between increased expression of these markers and Regimen 1 and Regimen 2 patients overall survival. More investigations of these findings will be the aim of future works when a custom made mRNA panel of genes will be run and more proteins will be stained in the associated TMA.

Overall these data clearly show how expression levels of miR-155, miR-493, miR-30e, miR-27a the 4 miRNAs belonging to the miRNAs signature, taken together, can serve as new diagnostic and prognostic factors in TNBC.

## DISCUSSION

Triple negative tumors exhibit a more aggressive phenotype and a worse clinical prognosis compared to other breast cancer subtypes. Only 30% of women with metastatic TNBC survive 5 years, and many patients eventually die of their disease. This is mainly determined by the high heterogeneity within these tumors and lack of definitive TNBC-specific therapeutic targets [2].Several studies have investigated the heterogeneity of TNBC from the molecular point of view. Cheang and colleagues first reported a significantly poorer survival in CB tumors compared to the 5NP subtype [2]. However, conflicting data have been reported [25, 26]. In this context, our group recently demonstrated that specific miRNA expression signatures characterize and contribute to the phenotypic diversity of TNBC. Moreover, miRNA expression profiles define different prognostic classes among TNBC patients [17].

Our current results further emphasize that the TNBC subtype includes distinctive cancer subtypes. In a series of 173 TNBCs, we tested the prognostic performances of both the 5 IHC markers panel and miRNA expression signatures. As reported previously by others [2, 3] we observed a poorer prognosis in CB patients in comparison to the 5NP group. Based on deregulated miRNA levels differentially expressed in the two different TNBC subtypes, a four miRNA signature (miR-155, miR-493, miR-30e, and miR-27a) was generated. This signature could discriminate among patients with high and low risk prognoses. As expected, low-risk tumors were highly enriched in 5NP samples.MiRNA profiling methods have been shown to be standardizable and of clinical impact in human cancers, even starting from routinely processed specimens. Moreover, miRNA expression profiling has been shown to be feasible and reliable also in cytological smears, the most common and least invasive method for breast cancer diagnosis. Altogether, such characteristics suggest miRNAs as suitable biomarkers to be introduced into clinical practice.

We identified a signature of four miRNAs that have been implicated in breast carcinogenesis. In particular, miR-27a and miR-155 deregulation has been demonstrated to significantly impact prognosis of breast cancer patients.

miR-27a indirectly regulates ER-alpha and hormone responsiveness in breast cancer-derived cell lines [27]. Moreover, it determines endothelial differentiation of breast cancer stem cells, promoting tumor neo-angiogenesis. Recently, miR-27a has been proposed as a novel marker of breast cancer progression and worse prognosis [28], which is in line with the observed up-regulation in CB samples in our series.

miR-155 has been implicated in tumor aggressiveness and resistance to chemotherapy *in vitro* and *in vivo*; its oncogenic role in leukemia and colon cancer has been previously established [29, 30]. Moreover, this miRNA has been confirmed as a novel plasma circulating biomarker of metastatic disease [31]. In TNBC, contrasting results have been produced [17, 32, 33]. In our series, miR-155 is significantly down-regulated in CB tumors, further supporting our seminal finding of its “protective” role in TNBC patients. How miR-155 exerts this caretaker role in TNBC is still under investigation and will be subject of future publications, we can anticipate that seems to be strictly correlated to the crucial role of DNA damage pathways in TNBC.

TNBC-specific targeted therapies remain undefined, and the most widespread approved therapeutic regimens are based on combinations of an anthracycline, a taxane, and/or an alkylating agent (typically cyclophosphamide) [12, 13, 34]. No validated biomarker is available to select patients with the highest benefit from the use of anthracycline, so far. In our series, CB subtype and high-risk patients (miRNA signature defined) had a better relative response to anthracycline-based therapies in comparison to 5NP, maybe due to the more aggressive molecular phenotype of the disease [35-37]. Our finding show the much better prognosis of the 5NP *vs* the CB cancers regardless of chemotherapy, as well as the lower efficacy of this therapy for the 5NP group; this subgroup need a much better targeted chemotherapy to increase overall survival.

Since patients treated with anthracycline alone had the best outcome compared to the combination of anthracycline + taxanes, we investigated possible taxanes chemoresistance pathways driven by miRNA-gene deregulation. Unfortunately the miRNA profile comparison between CB vs 5NP patients treated with Regimen 2 only due to the low cardinality of subcohort doesn't give statistically significant p-values (data not shown). Still, the interesting data about the lower overall survival of anthracycline + taxanes treated patients can be interpreted in different scenarios regardless of the group subdivision.

It is know that administration of neoadjuvant chemotherapy to breast cancer patients increases the fraction of CD44^+^/CD24^−/low^ tumor cells [38]. Connecting the deregulation of the BCSCs markers (CD44, CD24) to a possible selection of a more aggressive cancer phenotype [22, 24] by the chemotherapy Regimen 2, basically by the addition of taxanes, is an appealing scenario. Unfortunately in our cohort CD44^+^/CD24^−/low^ IHC data were available only for 6 cases treated with Regimen 1 and only for 4 patients treated with Regimen 2; these small numbers cannot give any trustworthy statistic or show any significant trend toward the involvement of BCSCs resistance in the observed inefficacy of the taxanes addition to the anthracycline regimen.

But if we consider only the CD44^+^/CD24^−/low^ patients and we stratified them in CB and 5NP, based on the status of the five IHC markers the overall survival curves are basically overlapping (Supplementary Figure 5A, p value is not statistically significant); while if we stratify those patients by miR-155, miR-493, miR-30e, miR- 27a expression levels, although again not statistically significant, the signature can divide the CD44^+^/CD24^−/low^ patients in high/low risk subgroups (Supplementary Figure 5B); opening to many different scenarios driven by miRNA deregulation.

Future development of this project will include the analysis of a custom made mRNA panel and more proteins assessment on the TMA. The detection of angiogenic factors and EMT/MET proteins known to be involved in the BCSC driven chemotherapy [24, 39] will hopefully help to clarify the involvement of BCSCs in TNBC.

The data shown so far support the conclusion that miRNAs are implicated in chemoresistance processes in breast cancers.

We can also analyze the contribution of non-stem cancer cells to the lower overall survival of Regimen 2 treated patients, focusing on miRNA targeted proteins. Of interest, taxanes-resistant cells display hallmarks of mesenchymal phenotype, including increased vimentin expression; its aberrant expression during EMT is suggested to be an essential element for epithelial plasticity and tumor cell metastasis [40, 41]. We can speculate that the worse outcome of CB cases treated with anthracycline + taxanes regimen can be due to the down-regulation of miR-30a (CB mean expression= 8.22, 5NP mean expression= 8.41) which will lead to increased vimentin expression in CB (CB, 43.5% positive versus 5NP, 27.9% positive p=0.044) which will induce taxane resistance as previously reported by Cheang et al.

Further studies should clinically validate such findings and test miR-30a impact on breast cancer therapeutic outcome.

In conclusion, we showed the importance of TNBC subclassification based on the 5 IHC markers method proposed by Cheang et al [3] and on a novel four miRNAs signature as new diagnostic tools. Both signatures have prognostic value, predicting patient outcomes based on different chemotherapy regimens. The development of new TNBC-targeted drugs should consider targeting the different TNBC subtypes as well as the miRNA-deregulated signal pathways in order to maximize patient outcome.

## METHODS

### Patients

An IRB-approved OSU protocol (Cancer Institutional Review Board of the Ohio State University) for this research linked clinical features, treatment and outcome data of breast cancer patients in the OSU National Comprehensive Cancer Network (NCCN) breast cancer database/tumor registry with archival breast cancer pathology specimens stored in the OSU Tissue Archive Service using the Information Warehouse at OSUMC to serve as “honest broker” and provided de-identified clinic-pathological information. No consent was required because the clinical data stored in the OSU Tumor Registry and pathologic specimens stored in Path Archives were de-identified. 365 consecutive TN localized breast cancer patients were identified from 1995-2005. After pathology review for tumors with sufficient sample for study, 173 paraffin blocks for TNBCs were identified for preparation of a tissue microarray and cores for RNA preparation, with the characteristics shown in the demographics summary in Table 1. For preparation of RNA, we used two 1.75 mm cores for tumor and for normal and two 0.6 mm cores were taken for preparation of the TMA in duplicate.

### Immunohistochemistry

Immunohistochemical reactions were obtained on 4 μm-thick sections and performed automatically (Dako Autostainer immunostaining system; Dako). Sections were then lightly counterstained with hematoxylin. Appropriate positive and negative controls were run concurrently. CK5/6 (D5/16 B4; Dako), EGFR (2-18C9; Dako) and Vimentin (M0275; Dako) immunostaining was performed on TMA sections.

The expression of CK5/6 and Vimentin was cytoplasmic, whereas the expression of EGFR was both cytoplasmic and membranous. Cytoplasmic expression in ≥10% of tumor cells for CK5/6, and membranous staining in ≥10% of tumor cells for EGFR were accepted as positive, as previously described. Expression of vimentin was scored as follows: sections were scored from 0–3 where ‘0’ corresponds to lack of positive staining and ‘3’ represents the most intense staining. Scores were calculated as follows: average intensity of the stain (1–3) x average percentage of positive cells, as previously described [42].

CD24 (SN3b; Neomarkers) and CD44 (DF1485; Dako) expression was determined by double staining of tumor cells [42, 43].

TNBCs were divided into subtypes of breast cancer as defined by their IHC profiles as basal-like triple negative (CB; negative for ER, PR, and HER-2 and positive for CK5/6 and/or EGFR), and five negative (5NP; negative for ER, PR, HER-2, CK5/6, and EGFR). Slides were scored independently by two pathologists (GG, SB) blinded to breast cancer subtype; one pathologist (MF) converted scores to numbers, selected cutoff values for each marker and entered data into Excel files.

### miRNA expression profiling and statistical analysis

Total RNAs were processed with the nanoString nCounter system (nanoString, Seattle, Washington, USA) in the Nucleic Acid Shared Resource of The Ohio State University. The miRNA panel detects 664 endogenous miRNAs (with 654 probes), 82 putative viral miRNAs, and 5 housekeeping transcripts. See Cascione et al [17] for expanded methods and validation of the microarray results.The miRNA microarray expression data have been submitted to the Gene Expression Omnibus (GEO) dataset with accession number GSE41970 (http://www.ncbi.nlm.nih.gov/geo/query/acc.cgi?acc=GSE41970).All fold-changes associated with these analyses are represented in log2 scale (logFC) and only data with a P-value (corrected for multiple comparisons by the Benjamini-Hochberg method) of ≤0.05 were considered significant.Hazard ratios (HR) were computed for a 2-fold change in the miRNA expression level. ‘Protective’ miRNAs were defined as those associated with an HR (from univariate Cox regression analysis) of less than one (HR<1); ‘risk-associated’ miRNAs were defined as those associated with an HR greater than one (HR>1).We estimated patient prognoses using Kaplan-Meier plots and the log-rank test. To generate a risk score, we adopted a previously developed strategy using the Cox regression coefficient of each dysregulated miRNA among 5NP and CB. The risk score for each patient was derived by multiplying the expression level of a miRNA by its corresponding coefficient. The patients were thus dichotomized into groups at high or low risk using the 50th percentile (median) cutoff of the risk score as the threshold value.

### Validation (from Cascione et al. 2013)

To validate these study findings, three approaches were used: first we validated the deregulated miRNAs “*in silico*” using the database published by Farazi et al 2011 [44] [GEO: GSE28884]. From the analysis of this database, based on an Illumina Genome Analyzer IIx deep sequencing platform, we were able to confirm the expression pattern of the 69% of our miRNAs cohort represented among the sequences.

The Cancer Genome Atlas (TCGA) [45] dataset, also based on deep sequencing, was used for a second “*in silico*” validation, here the expression pattern of the whole miRNA profile was confirmed by the 73%.

In a subset of samples (randomly chosen based on availability of RNAs) we were able to validate the expression levels of a subset of miRNAs (7 differentially expressed miRNAs shown in Supplementary Figure S1, plus 2 miRNAs used as normalizers) by TaqMan® qRT-PCR assay. Box plots representing this qRT-PCR based validation are shown in Supplementary Figure S3. Absence of undetermined values in the Real-Time raw data (not shown) also indicates low levels of RNA degradation, a concern when FFPE samples are involved.

An external TNBC cohort of 48 FFPE tissues was considered. This second cohort was also profiled by the nanoString nCounter method and the miRNA profiles were analyzed following the criteria for the previous cohort; results of study of this independent cohort confirmed the deregulation of the 79% of the miRNAs observed to be dysregulated in the current study.For a subset of samples belonging to this independent cohort (20 samples), the 5 markers IHC data were also available. We sub-typed patients into CB and 5NP and to proceed with the validation of the OS based on IHC and miRNA signature status (Supplementary Figure S4). The diagnostic value of the TNBC subtyping based on IHC and miRNA signature was validated by OS as shown in Supplementary Figure S4A and Supplementary Figure S4B.From analysis of the dataset associated with this independent cohort, the diagnostic value of both the signatures couldn't be validated due to the small number of events within the 20 samples (only 4).

## SUPPLEMENTARY FIGURES AND TABLES




