# Supplementary material for: microRNA expression profiling identifies a four microRNA signature as a novel diagnostic and prognostic biomarker in triple negative breast cancers
Source: Oncotarget. 2014 Jan 21;5(5):1174–84. doi: 10.18632/oncotarget.1682 (PMC4012726; doi:10.18632/oncotarget.1682)
Supplement: Supplementary file 1 [file oncotarget-05-1174-s001.pdf]

microRNA expression profiling identifies a four microRNA signature as a novel diagnostic and prognostic biomarker in triple negative breast cancers – Gasparini et al

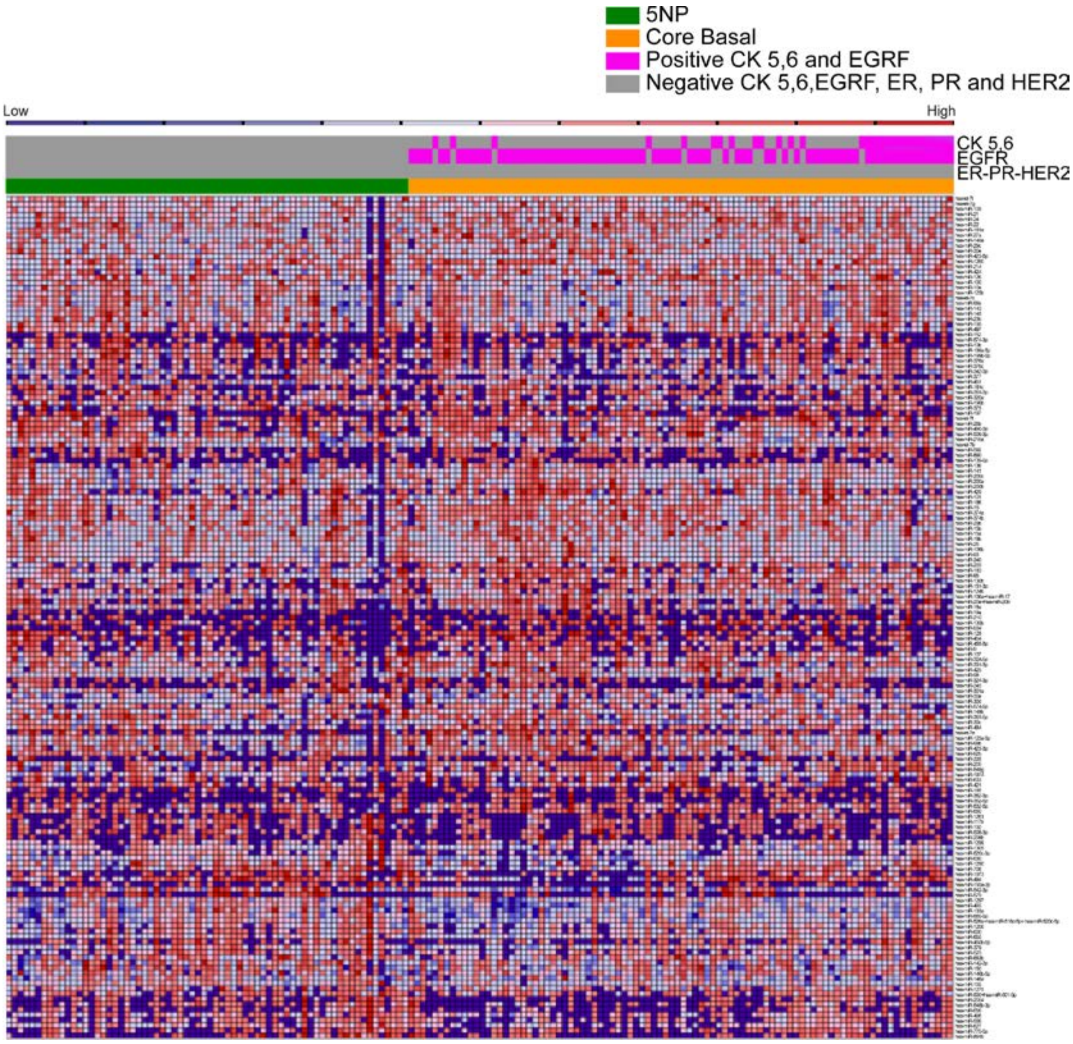

SUPPLEMENTARY FIGURE 1. Heat map of miRNAs expressed in the CB and 5NP TNBCs. Heat map representing unsupervised hierarchical clustering for 160 clinical samples, 161 miRNAs expressed in tumors are shown. On top of the heatmap the color coded bars identify the 5 IHC markers results for each patient and the distinction between CB and 5NP. Samples are shown in columns, miRNAs in rows. Heat map colors represent relative miRNA expression as indicated in the blue to red key bar at the top

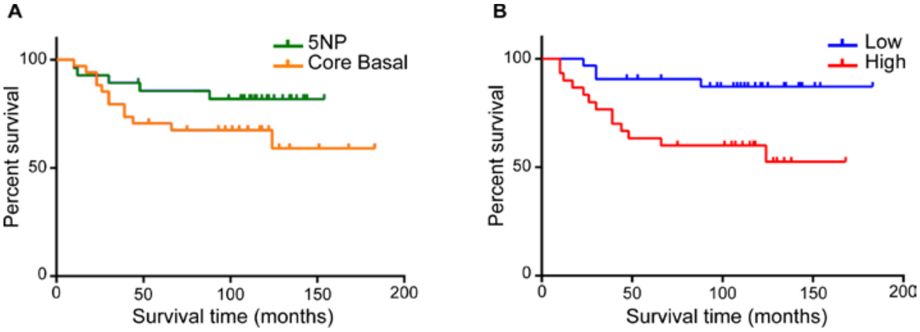

SUPPLEMENTARY FIGURE 2 (A, B). Overall Survival of patients for whom chemotherapy treatment information was not available (NA). **A)** Overall survival of patients we don't have info about the chemotherapy regimen (NA), stratified by 5 IHC markers status. **B)** COX proportional hazard model of unknown chemotherapy regimen treatment, stratified by high/low risk 4 miRNA signature.

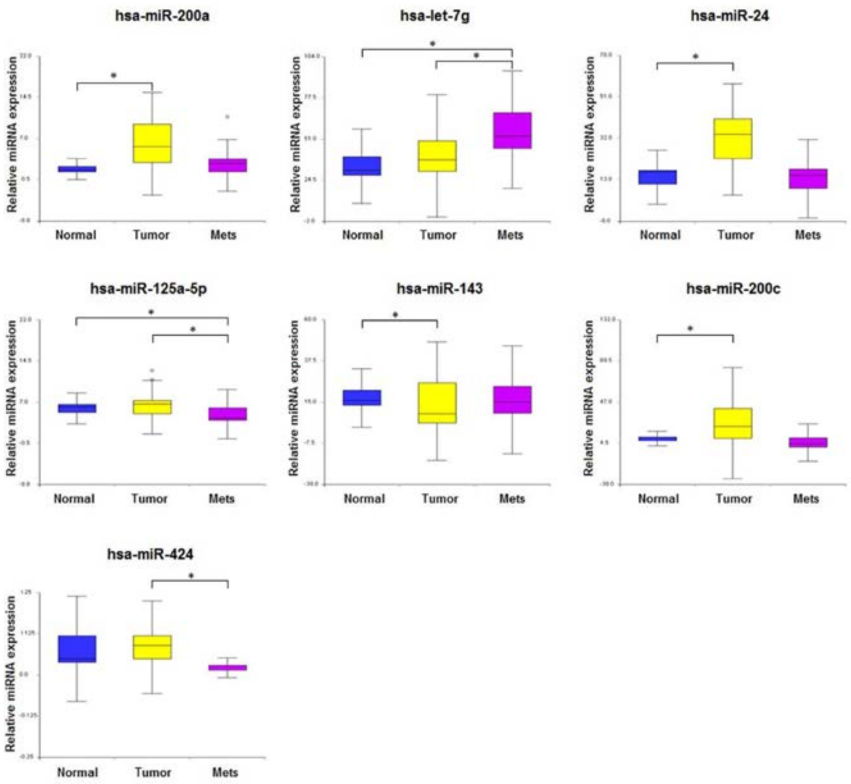

SUPPLEMENTARY FIGURE 3. qPCRs expression data of deregulated miRNAs in the original TNBC cohort (from Cascione et al 2013 supporting information). Box plots represent expression of the miRNAs in representative samples of the three tissue groups, assayed by TaqMan® qPCR. Results are represented as $2^{-\Delta Ct}$ relative expression to RNU6B. Error bars ± s.d., $^{*}P < 0.05$, by two-tailed Student's t test.

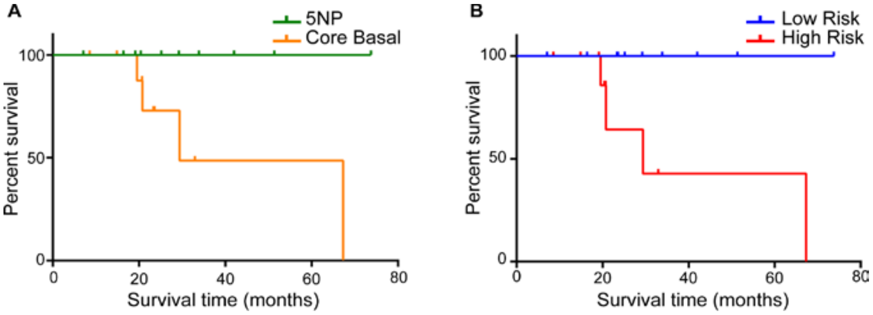

SUPPLEMENTARY FIGURE 4 (A, B). Diagnostic validation in external cohort. **A)** Overall Survival based on the status of the five IHC markers. **B)** Overall survival based on the four miRNA signature.

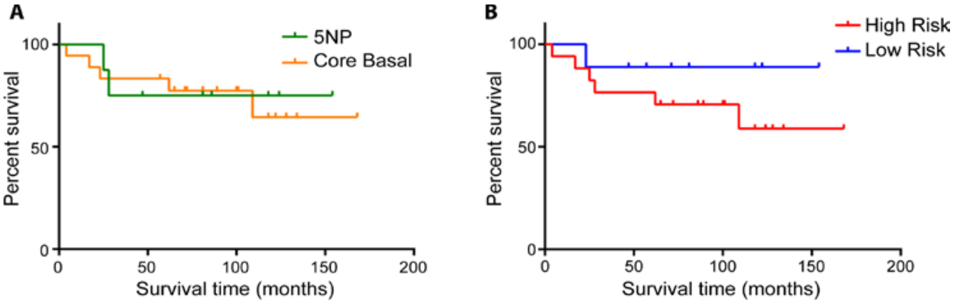

SUPPLEMENTARY FIGURE 5 (A, B). Overall survival of $CD44^{+}$/$CD24^{-/low}$ TNBC patients. **A)** Overall Survival of $CD44^{+}$/$CD24^{-/low}$ based on the status of the five IHC markers. **B)** Overall survival of $CD44^{+}$/$CD24^{-/low}$ based on the four miRNA signature, both are statistically not significant.
