# Supplementary material for: microRNA expression profiling identifies a four microRNA signature as a novel diagnostic and prognostic biomarker in triple negative breast cancers
Source: Oncotarget. 2014 Jan 21;5(5):1174–84. doi: 10.18632/oncotarget.1682 (PMC4012726; doi:10.18632/oncotarget.1682)
Supplement: Supplementary file 2 [file oncotarget-05-1174-s002.pdf]

Supplementary Table 1
Top 4 deregulated miRNAs in the comparison between CB and 5NP breast cancers

microRNA ID	Core Basal (CB) mean	Five Negative (5NP) mean	log2FC CB vs 5NP	Adj-p-values CB vs 5NP
**hsa-miR-155**	6.23	6.99	0.76	0.04
**hsa-miR-493**	6.47	7.01	0.54	0.01
**hsa-miR-30e**	7.85	7.24	-0.61	0.04
**hsa-miR-27a**	8.58	7.78	-0.80	0.01

Supplementary Table 2
Hazard ratios of protective and risky miRNAs

microRNA ID	Hazard Ratio	Type
**hsa-miR-155**	0.73	Protective
**hsa-miR-493**	0.88	Protective
**hsa-miR-30e**	1.08	Risky
**hsa-miR-27a**	1.09	Risky
